# Piezoelectric rubber sheet sensor: a promising tool for home sleep apnea testing

**DOI:** 10.1007/s11325-024-02991-9

**Published:** 2024-02-15

**Authors:** Junichiro Hayano, Hiroaki Yamamoto, Haruhito Tanaka, Emi Yuda

**Affiliations:** 1 Heart Beat Science Lab Inc., Sendai, Japan; 2 Gifu Mates Sleep Clinic, Gifu, Japan; 3 International Institute for Integrative Sleep Medicine (IIIS), University of Tsukuba, Tsukuba, Japan; 4 Graduate School of Information Sciences, Tohoku University, Sendai, Japan

**Keywords:** Sleep apnea, Micromotion, Piezoelectric rubber sheet sensor, Respiratory event index, Home sleep apnea testing

## Abstract

**Purpose:**

This study aimed to develop an unobtrusive method for home sleep apnea testing (HSAT) utilizing micromotion signals obtained by a piezoelectric rubber sheet sensor.

**Methods:**

Algorithms were designated to extract respiratory and ballistocardiogram components from micromotion signals and to detect respiratory events as the characteristic separation of the fast envelope of the respiration component from the slow envelope. In 78 adults with diagnosed or suspected sleep apnea, micromotion signal was recorded with a piezoelectric rubber sheet sensor placed beneath the bedsheet during polysomnography. In a half of the subjects, the algorithms were optimized to calculate respiratory event index (REI), estimating apnea–hypopnea index (AHI). In the other half of subjects, the performance of REI in classifying sleep apnea severity was evaluated. Additionally, the predictive value of the frequency of cyclic variation in heart rate (Fcv) obtained from the ballistocardiogram was assessed.

**Results:**

In the training group, the optimized REI showed a strong correlation with the AHI (*r* = 0.93). Using the optimal cutoff of REI ≥ 14/h, subjects with an AHI ≥ 15 were identified with 77.8% sensitivity and 90.5% specificity. When applying this REI to the test group, it correlated closely with the AHI (*r* = 0.92) and identified subjects with an AHI ≥ 15 with 87.5% sensitivity and 91.3% specificity. While Fcv showed a modest correlation with AHI (*r* = 0.46 and 0.66 in the training and test groups), it lacked independent predictive power for AHI.

**Conclusion:**

The analysis of respiratory component of micromotion using piezoelectric rubber sheet sensors presents a promising approach for HSAT, providing a practical and effective means of estimating sleep apnea severity.

## Introduction

Home sleep apnea testing (HSAT) [1], as a means of screening sleep apnea, plays a crucial role in efficiently utilizing the limited medical resources of polysomnographic examination. Various types of sensors are utilized in HSAT devices, including those for measuring nasal pressure/temperature [2], respiratory inductance plethysmography [3], peripheral arterial tonometry [4, 5], oximetry [6], electrocardiography [7, 8], pulse wave photo-plethysmography [9], radio-wave Doppler effect [10] and bed-embedded micromotion sensors [11–14]. Generally, it is believed that the accuracy of sleep apnea detection improves with an increased number of signals measured [15]. However, considering the convenience of HSAT, it is desirable to minimize the number of sensors used and the effort required to wear them [16]. Thus, the optimal signal and measurement method should be selected by considering the tradeoff between accuracy and convenience.

Among the sensors utilized in HSAT, micromotion sensing by sheet sensors positioned beneath the bedsheet presents the notable advantage of enabling users to sleep without the need for wearing sensors or electrodes while remaining unaware of their presence. Additionally, these sensors possess a unique feature of capturing respiration and ballisttocardioram as well as body movement using a single sensor device [13]. Thus, they could be a promising solution for HSAT, effectively meeting both accuracy and convenience requirements simultaneously. In this study, we developed an unobtrusive method for HSAT utilizing a piezoelectric rubber sheet sensor. We designated algorithms to extract respiration and ballistocardiogram signals, allowing for the scoring of the respiratory event index (REI) [16, 17], as well as measuring the frequency (Fcv) of cyclic variation of heart rate (CVHR) [7, 8, 18]. The performance of REI and Fcv in classifying sleep apnea severity was assessed using the apnea–hypopnea index (AHI) obtained from the simultaneous polysomnogram as a reference standard.

## Methods

### Ethics approval and consent to participate

All procedures were performed in accordance with the protocol that was approved by the Research Ethics Committee of the Center for Data-driven Science and Artificial Intelligence, Tohoku University, Japan (registration number 2022–7). All subjects participated in this study gave their written informed consent.

### Subjects

The eligible subjects for this study were patients who underwent an overnight polysomnography due to suspected or diagnosed sleep disordered breathing at Gifu Mates Sleep Clinic in Gifu, Japan, between September 2022 and December 2022. The inclusion criterion was adulthood (age 20 years or older). Subjects were excluded if they had continuous atrial fibrillation, experienced acute illness, or had exacerbation of chronic diseases requiring hospitalization within the past three months. Additionally, individuals who were pregnant or breastfeeding were also excluded.

### Protocol

The polysomnographic examination was performed with an Alice diagnostic sleep system (Philips-Respironics, Murrysville, PA, USA Philips Respironics, The Netherlands). The examination was initiated at the subject's customary bedtime and continued until the subject awoke the next morning, during which micromotions were continuously measured by a commercially available piezoelectric rubber sheet sensor device (Moni Life wellness®, Sumitomo Riko Company Limited, Komaki, Aichi, Japan).

Subjects were randomly allocated into a training group and a test group. Using the data from the training group, we developed and optimized the algorithms for sleep apnea detection, constructed regression models to estimate sleep apnea severity, and identified the optimal cutoff values for classifying the severity. Using the data from the test group, we evaluated the classification performance of the algorithms.

### Measurements

The polysomnograms were recorded with the standard montages consisting of F4-M1, F4-M2, C4-M1, C3-M2, O2-M1, and O1-M2 electroencephalograms, left and right electrooculograms, a submental electromyogram, a nasal pressure cannula, oronasal airflows, left and right tibial electromyograms, thoracoabdominal inductance plethysmograms, pulse oximetric arterial blood oxygen saturation, a neck microphone, body position sensors, and a modified lead II ECG.

Sleep stages, apneic and hypopneic indices (AI and HI, respectively), and AHI were scored according to the American Association of Sleep Medicine (AASM) Manual for the Scoring of Sleep and Associated Events, Version 2.5 [19] by registered polysomnogram technicians. AHI was calculated both with the total recording time (TRT) as the denominator (AHI_TRT_) and total sleep time (TST) as the denominator (AHI_TST_). The AHI_TRT_ was used as the reference standard for developing algorithms for sleep apnea detection from the micromotion signal. The AHI_TST_ was used to classify sleep apnea severity, with < 5 defined as normal, 5–15 as mild, 15–30 as moderate, and ≥ 30 as severe sleep apnea. The ECG signal of the polygraph was sampled at a frequency of 100 Hz. All QRS complexes were identified and annotated as normal (sinus rhythm), ventricular ectopic beat, supraventricular ectopic beat, and artifact.

**Fig. 1:**
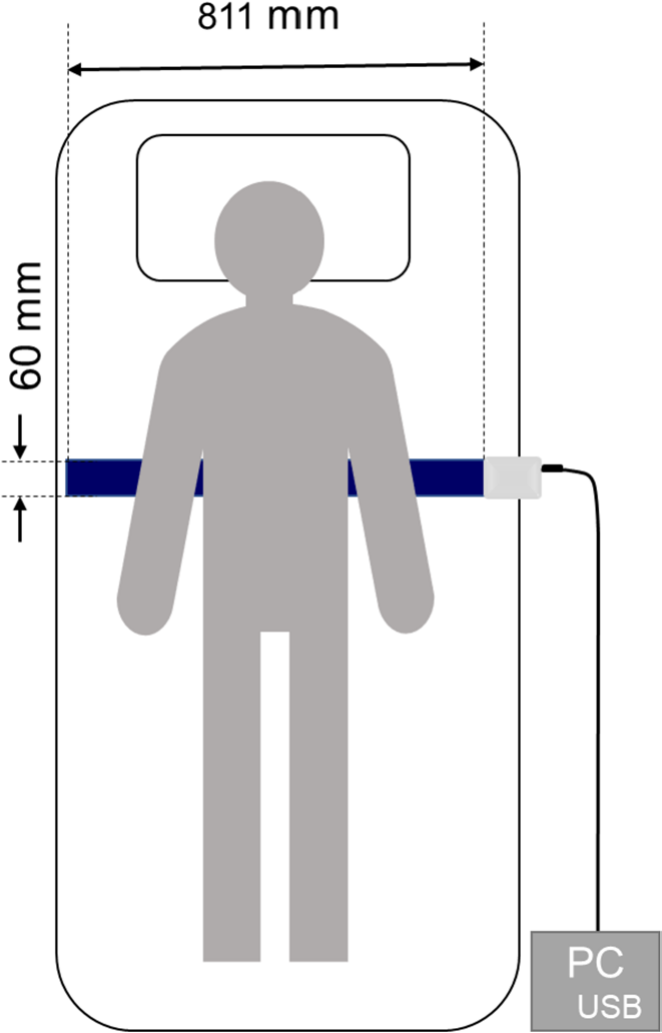
Schema of piezoelectric rubber sheet sensor used for measuring micromotion

The piezoelectric rubber sheet sensor, depicted in Fig. 1, had dimensions of 811 mm in length, 60 mm in width, and 0.9 mm in thickness and a weight of 220 g. It was positioned beneath the bedsheet at a level from the subject's axilla to the lower end of the sternum body. The signal was digitized at 100 Hz with a 24-bit dynamic range (± 2^23^, from -8,388,608 to + 8,388,608) and the device outputted the data to a CSV file.

### Development of REI algorithms

**Fig. 2:**
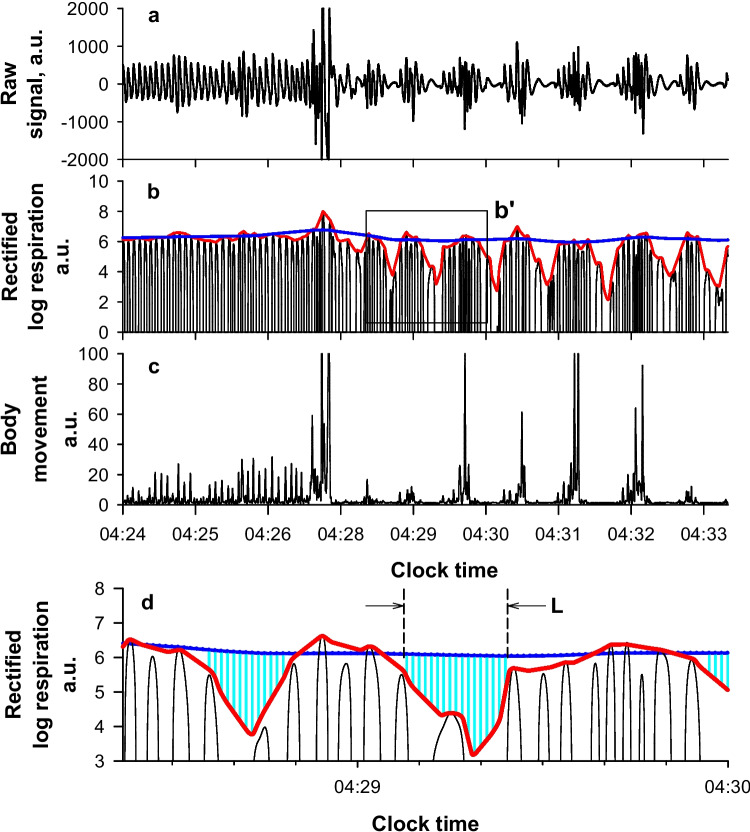
Detection of sleep apnea by micromotion signal from piezoelectric rubber sheet sensor. A series of sleep apnea attacks began to appear at 04:28, with the fast upper envelope of respiration (red line in panel b) declining from the slow upper envelope (blue line in panel b) and showing periodic dips. Panel d is the enlarged image of frame b’ in panel b. Periods in which the dip of the fast upper envelope (red line) from the slow upper envelope (blue line) exceeds a depth threshold (ln (1.43), which corresponds to a 30% drop) are indicated by vertical lines (cyan). When the length (L) of a period meets duration criteria (10 to 70 s), the period is considered to be a respiratory event

The details of the algorithms are reported in Appendix. Briefly, first, the algorithm extracted respiratory, body movement, and ballistocardiogram components from the micromotion signal of the piezoelectric rubber sheet sensor using band-pass filters set to their respective frequency ranges (0.08–0.5, 2–3, and 4–11 Hz) (Fig. 2). Second, the respiratory component signals were rectified to reflect the magnitude of respiratory motion and natural logarithm transformation was performed to minimize the effects of large changes in respiratory amplitude and large noise. Third, two types of upper (95th percentile point) envelopes (fast and slow) were generated; the fast envelope depicted breath-by-breath amplitude changes, while the slow envelope presented the local trend of submaximum amplitude. The use of the 95th percentile points for the envelopes excluded outliers with an incidence < 5%. Fourth, periods in which the fast envelope separated downward from the slow envelope to an extent greater than a threshold (depth threshold) were detected. Fifth, when the length of a period was within a range (duration criteria), the period was considered to be a respiratory event. Finally, REI was calculated as the frequency of respiratory events per hour of TRT.

The optimal values of depth threshold and duration criteria were determined in the training group using a grid-search method. This involved repeated analyses with small parameter adjustments until the closest correlation between REI and AHI was achieved. Because the amplitude of respiratory movement detected by the sheet sensor varied substantially with body position, a percentage reduction was employed as the depth threshold to adapt to the variations. The search for the optimal depth threshold was conducted in 1% increments, while the optimal lower and upper duration criteria were determined at 10-s intervals.

The source code for these algorithms was written in FORTRAN 95 and compiled on Microsoft Windows 10 by the Silverfrost Fortran (FTN95) compiler (Elgin, IL, USA).

### Analysis of Fcv

Fcv was measured not only from the heartbeat interval of the ballistocardiogram but also from ECG R-R interval of the polysomnography (ECG-Fcv). The method for measuring the heartbeat interval from ballistocardiogram is reported in Appendix. CVHR was detected by previously published algorithm [7, 8, 18] and Fcv and ECG-Fcv was computed as the frequency of CVHR per hour of TRT. The ECG-Fcv was used to determine whether the association between ballistocardiogram Fcv and AHI_TRT_ is affected by the accuracy of ballistocardiogram-based heartbeat interval measurement.

### Statistical analysis

The statistical analyses were performed using the program package of Statistical Analysis System (SAS Institute, Cary, NC, USA). Between-group differences in quantitative and categorical variables were assessed using the Wilcoxon rank sum test and χ^2^ test, respectively. The relationships between AHI_TRT_ and REI, Fcv, and ECG-Fcv were evaluated with Pearson's correlation coefficient. The multivariate regressions were performed using the SAS REG procedure. The classification performance of REI, Fcv, and ECG-Fcv for binary sleep apnea severity was evaluated by the area under the curve (AUC) of the receiver-operating characteristic (ROC) curve. The classification performance for four severity levels (normal, mild, moderate, and severe) was examined by the percentages of subjects correctly classified and misclassified off by one, two, and three classes. The optimal REI cutoff values for these classifications were determined by balancing sensitivity and specificity by ROC curve analysis in the training group and then evaluated in the test group. Statistical significance was defined as *P* < 0.05.

## Results

### Subjects’ characteristics

We enrolled a total of 78 consecutive subjects (21 females) with a median (interquartile range, IQR) age of 49 (38–62) years who underwent polysomnography for diagnostic purposes (*n* = 54, 69%) or evaluation of therapeutic effects (*n* = 24, 31%). The polysomnography revealed that the median (IQR) AHI_TST_ was 13.4 (3.6 to 23.8), with 34 (44%) subjects classified as having moderate-to-severe sleep apnea (AHI_TST_ ≥ 15) and 18 (23%) subjects classified as having severe sleep apnea (AHI_TST_ ≥ 30). Among apnea episodes, obstructive, central, and mixed types accounted for 72%, 4%, and 24%, respectively. Half (*n* = 39) of the subjects were assigned to the training group and the other half (*n* = 39) to the test group. There were no significant differences in the characteristics of the subjects between the two groups (Table 1).

**Table 1:** Subjects’ characteristics in the training and test groups

	Training group	Test group	P*
	*N* = 39	*N* = 39	
Age, year	55 (42–67)	45 (36–52)	0.5
Female, n (%)	11 (28%)	10 (26%)	0.7
BMI, kg/m2	24.8 (22.9–28.7)	25.3 (21.6–30.8)	0.4
*Purpose of polysomnography*			0.5
Diagnosis, n(%)	26 (66%)	28 (71%)	
CPAP titration, n(%)	10 (26%)	10 (26%)	
Other, n(%)	3 (8%)	1 (3%)	
Total recording time, min	474 (447–485)	467 (458–482)	0.2
Total sleep time, min	369 (317–417)	406 (356–424)	0.3
Sleep efficiency, %	82.7 (70.6–87.4)	85.3 (76.7–91.8)	0.7
AHI_TST_	13.9 (4.1–47.4)	12.9 (2.8–23.8)	0.5
AI_TST_	1.4 (0.3–9.5)	1.2 (0.3–6.2)	0.6
HI_TST_	7.2 (2.7–16.8)	10.8 (2.8–17.4)	0.7
OAI_TST_	0.8 (0.0–6.5)	0.3 (0.0–6.2)	0.7
CAI_TST_	0.2 (0.0–0.4)	0.1 (0.0–0.6)	0.3
MAI_TST_	0.2 (0–0.5)	0.1 (0–0.5)	0.5
AHI_TST_ ≥ 15	18 (46%)	16 (41%)	0.6
AHI_TST_ ≥ 30	10 (26%)	8 (21%)	0.5
PLM index ≥ 15	1 (3%)	2 (5%)	0.5

Data are median (IQR) or frequency (%)

^*^Significance of difference by Wilcoxon rank sum test

AHI = apnea–hypopnea index, AI = apnea index, CAI = central apnea index, HI = hypopnea index, BMI = body mass index, CPAP = continuous positive airway pressure, MAI = mixed apnea index, OHI = obstructive apnea index, PLM = periodic leg movement. The denominator for AHI, AI, HI, OAI, CAI, and MAI is TST (in hour)

### Grid search for optimal parameters

In the training group, the grid search was conducted to determine the optimal parameter values. The results revealed that the closest correlation between REI and AHI_TRT_ was achieved when the depth threshold was set at 30% (equivalent to a natural-logarithmic transformed amplitude difference of ln (1.43)) and the duration criteria was configured to select dips with a length between 10 and 70 s as respiratory events. These parameters were used for analyzing the data in the test group.

### Estimation of AHI_TRT_ by REI and Fcv

**Fig. 3:**
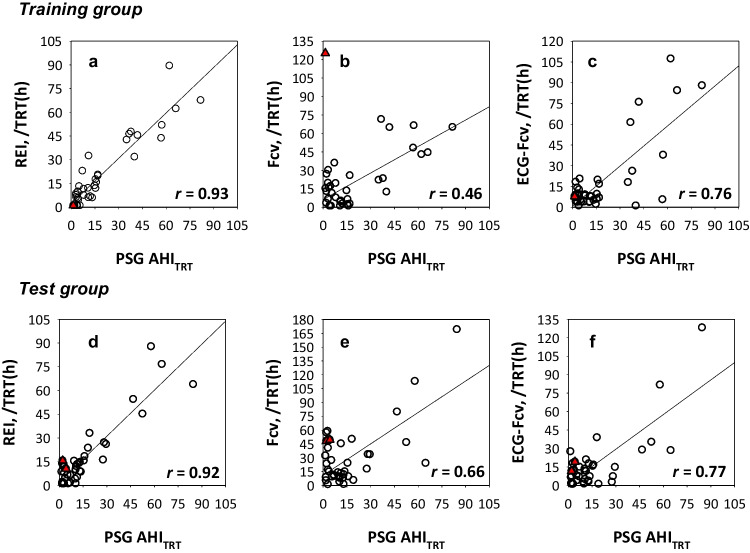
Correlation of alternative measures of AHI with the true measure in the training group (panels a-c) and test group (panels d-f)**.**  Subjects with a PLM index ≥ 15. AHI = apnea–hypopnea index, ECG = electrocardiogram, Fcv = frequency of cyclic variation of heart rate, PLM periodic leg movement, PSG = polysomnography, REI = respiratory event index, TRT = total recording time

**Fig. 4:**
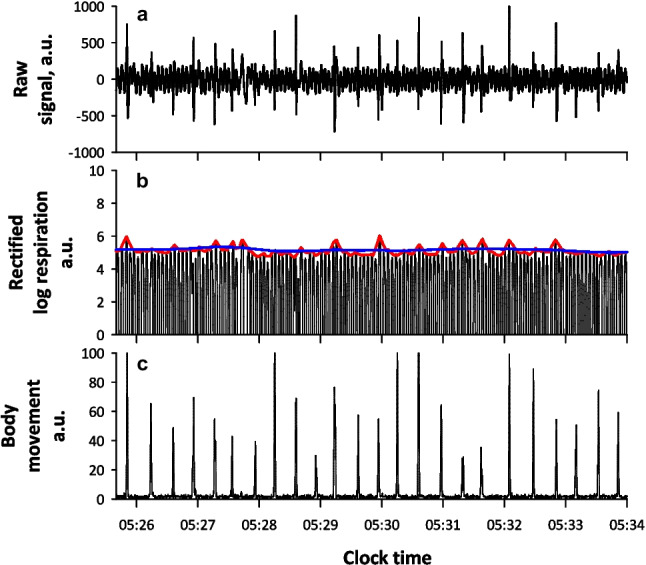
Micromotion signal from piezoelectric rubber sheet sensor and extracted respiratory and body movement signals during an event of periodic leg movement in a subject with a periodic leg movement index of 108.8

In the training group, the REI calculated with the optimized parameters closely correlated with AHI_TRT_ (r = 0.93), while the Fcv and ECG-Fcv correlated with correlation coefficients of 0.46 and 0.76, respectively (Fig. 3). When applying the same algorithms and parameters to the data in the test group, the correlation coefficients became 0.92, 0.66, and 0.77 for REI, Fcv, and ECG-Fcv, respectively. Notably, in both the training group and test group, the correlations of Fcv were lower compared to those of REI, as well as the correlations of ECG-Fcv. These differences were partly attributed to the impact of PLM on Fcv (Figs. 3 and 4). When excluding subjects with a PLM index of ≥ 15, the correlation coefficients of REI, Fcv, and ECG-Fcv became 0.92, 0.68, and 0.76, respectively, in the training group and 0.91, 0.70, and 0.79, respectively, in the test group.

Multiple regression analyses were performed to assess the relationship between AHI_TRT_ and the combination of REI and Fcv in the training and test groups. In either group, the inclusion of Fcv lead to no significant improvement in the multiple correlation when REI was already included, even when the subjects with PLM index of ≥ 15 were excluded.

### Sleep apnea severity classification by REI

Table 2 displays the results of the ROC curve analysis evaluating the classification performance of sleep apnea severity using the REI, Fcv, and ECG-Fcv. In both the training and test groups, the REI demonstrated favorable classification performance, as indicated by AUC values exceeding 0.9, for both moderate-to-severe sleep apnea and severe sleep apnea. The AUC values of REI were higher than those of Fcv for both levels of severity and those of ECG-Fcv for severe sleep apnea.

**Table 2:** Receiver operating characteristic curve analysis of sleep apnea severity classification performance by REI

	*N*		AUC (95%CI)			Difference, P*	
	Yes	No	REI	Fcv	ECG-Fcv	REI vs ECG-Fcv	REI vs Fcv
*Training group*							
AHI_TST_ ≥ 15	18	21	0.907 (0.771–0.976)	0.685 (0.517–0.824)	0.694 (0.527–0.832)	0.02	0.01
AHI_TST_ ≥ 30	10	29	0.997 (0.903–1.00)	0.866 (0.718–0.953)	0.826 (0.671–0.928)	0.1	0.03
*Test group*							
AHI_TST_ ≥ 15	16	23	0.937 (0.811–0.99)	0.614 (0.445–0.765)	0.682 (0.514–0.822)	0.004	0.0003
AHI_TST_ ≥ 30	8	31	0.980 (0.874–1.00)	0.839 (0.686–0.937)	0.782 (0.621–0.898)	0.05	0.02

^*^Significance of difference of the AUC of REI

The ROC curve analysis in the training group revealed that REI ≥ 14 and REI ≥ 24 were optimal cutoff values for detecting moderate-to-severe sleep apnea and severe sleep apnea, respectively (Table 3). When applying these cutoff values to the data in the test group, 87.5% sensitivity and 91.3% specificity for moderate-to-severe sleep apnea and 87.5% sensitivity and 96.8% specificity for severe sleep apnea were obtained (Table 3).

**Table 3:** Confusion table of sleep apnea severity classification by REI in the training and test groups

Training group				
	Cutoff	AHI_TST_ ≥ 15	AHI_TST_ < 15	
REI	≥ 14	14	2	PPA = 87.5%
	< 14	4	19	NPA = 82.6%
		Sensitivity = 77.8	Specificity = 90.5%	Accuracy = 84.6%
	Cutoff	AHI ≥ 30	AHI < 30	
REI	≥ 24	10	1	PPA = 90.9%
	< 24	0	28	NPA = 100.0%
		Sensitivity = 100%	Specificity = 96.6%	Accuracy = 97.4%
Test group				
	Cutoff	AHI_TST_ ≥ 15	AHI_TST_ < 15	
REI	≥ 14	14	2	PPA = 91.3%
	< 14	2	21	NPA = 87.5%
		Sensitivity = 87.5%	Specificity = 91.3%	Accuracy = 87.2%
	Cutoff	AHI ≥ 30	AHI < 30	
REI	≥ 24	7	1	PPA = 87.5%
	< 24	1	30	NPA = 96.8%
		Sensitivity = 87.5%	Specificity = 96.8%	Accuracy = 94.9%

Finally, in the training group, the most favorable outcome for the classification of severity into four levels was achieved by incorporating cutoff values of REI < 9 to define the normal category, and 9 to 14 to define mild sleep apnea (Table 4). Subsequently, When applying these identical cutoff values to the test group, the classification accuracy reached 56.4% and 82% of the misclassifications were off by one class and the rest (18%) were off by two classes (Table 4).

**Table 4:** Four-level severity classification of sleep apnea by REI in the training group

		AHI_TST_			
		< 5 Normal	5–15 Mild	15–30 Moderate	≥ 30 Severe
*Training group*					
REI	< 9	12	3	2	0
	9–14	1	3	2	0
	14–24	0	1	4	0
	≥ 24	0	1	0	10
Sensitivity, %	92	38	50	100	92
Specificity, %	81	90	97	97	81
*Test group*					
REI	< 9	7	7	2	0
	9–14	4	3	0	0
	14–24	1	1	5	1
	≥ 24	0	0	1	7
Sensitivity, %	58	27	63	88	58
Specificity, %	67	86	90	97	67

For the training group, accuracy (correct classification ratio) is 74.4% and misclassification off by 1 and 2 classes are 70% and 30%, respectively. For the test group, accuracy (correct classification ratio) is 56.4% and misclassification off by 1 and 2 classes are 82% and 18%, respectively. The sensitivity in the table is the proportion of subjects in the AHI class who were classified in that class, and the specificity is the proportion of subjects not in the AHI class who were identified as not in that class

## Discussion

To develop an unobtrusive method for HSAT utilizing micromotion signals detected by a piezoelectric rubber sheet sensor, we developed algorithms to extract respiratory and ballistocardiogram components from these signals and detect respiratory events. In a group of 78 adult subjects with diagnosed or suspected sleep apnea, data from half of the subjects were used to optimize algorithms to calculate REI estimating AHI_TRT_, while the other half was used to evaluate REI's performance in classifying sleep apnea severity. Additionally, the predictive value of Fcv from the ballistocardiogram was assessed. The optimized REI closely correlated with AHI, effectively identifying subjects with AHI ≥ 15 with high sensitivity and specificity in both training and test groups. However, while Fcv showed a modest correlation with AHI, it lacked independent predictive power. These results suggest that analyzing the respiratory component of micromotion using piezoelectric rubber sheet sensors offers a promising and practical avenue for HSAT, providing an effective means of estimating sleep apnea severity.

Although the analysis of micromotion signals has garnered increasing attention as a noninvasive method for obtaining respiration and heartbeat signals during sleep [11–14, 20], the use of these signals requires addressing two crucial challenges: the frequent inclusion of substantial noise, even with minimal body movements, and the notable variability in the magnitude of the respiratory signal with changes in body posture and position. The REI assessment algorithms developed in this study addressed these issues by a method utilizing the fast and slow envelopes of the 95th percentile points of logarithmically transformed respiratory amplitude. By employing the 95th percentile points for the envelopes, the influences of outliers with an incidence < 5% were excluded. Moreover, the logarithmic transformation minimized extensive deviations of the envelopes caused by large noise. As sleep apnea–hypopnea events were identified based on the relative percent reduction in the fast envelope compared to the slow envelope, the detection threshold automatically adapted to variations in respiratory amplitude reflected in the slow envelope. With these features, the algorithms provided an optimized REI that identified subjects with an AHI ≥ 15 with a sensitivity of 87.5% and specificity of 91.3% in the test group. This classification performance compared favorably even with the performance of Type 3 HSAT devices studied in the American Academy of Sleep Medicine's clinical guidelines for sleep apnea diagnosis (six devices studied in 457 participants, with sensitivity ranging from 62 to 94% and specificity ranging from 25 to 97%) [21].

In previous studies investigating sleep apnea–hypopnea detection through micromotion, these challenges were addressed using diverse approaches. Agatsuma et al. [11] evaluated the performance of a sheet sensor, SD-101, for HSAT. The SD-101 consisted of 162 membrane-type pressure sensors arranged on a sheet 1235 mm long, 555 mm wide, and 5 to 7 mm thick. The device had the ability to automatically find and select one of the 162 sensors that most significantly sensed respiratory motion and had the least noise. In 201 patients with suspected sleep apnea, the REI measured by the SD-101 correlated with AHI_TRT_ (*r* = 0.88); REI ≥ 14 identified patients with AHI_TST_ ≥ 15 with 89.5% sensitivity and 85.8% specificity. Their findings were replicated by Kobayashi et al. [22], with a correlation coefficient of 0.87 and a sensitivity of 87.5% and specificity of 85.7% for identification of patients with AHI_TST_ ≥ 15. Sadek et al. [13] conducted a study using a microbend fiber optic sensor mat measuring 20 cm × 50 cm × 0.5 cm in ten patients with obstructive sleep apnea. They devised an adaptive histogram-based thresholding approach for detecting respiratory events. This method involved creating a histogram from the absolute deviations of respiratory signal in overlapping 60-s segments and determined the optimal cutoff to detect segments with a respiratory event. Using this approach, they achieved a sensitivity of 57.07% and a specificity of 45.26% in detecting true individual respiratory events. Coluzzi et al. [14] developed a multi-scale algorithm for detecting sleep fragmentation. The algorithm analyzed the cumulative histogram of quiet sleep segment lengths derived from micromotion signals obtained by a pressure bed sensor measuring 64 cm × 64 cm. They reported that the ratio between the total fragmented sleep time and the total moving time had a correlation coefficient of 0.85 with AHI in 18 subjects who underwent polysomnography. Finally, Weinreich et al. [20] conducted a study in 57 patients with obstructive sleep apnea or PLM using Sleep Minder, a radio wave Doppler sensor, that enabled non-contact micromotion sensing. The detection algorithm employed by the device was not disclosed. While the AHI estimated by this device had a correlation coefficient of 0.57 with the true AHI, it displayed a correlation coefficient of 0.79 with the sum of the true AHI and PLMI by the polysomnography. Moreover, it successfully identified patients with a sum of AHI + PLMI ≥ 15/h with a sensitivity of 92.2% and a specificity of 95.8%. Due to differences in device type, study subjects, and taction target, comparing the performance of algorithms is not feasible. Nonetheless, the algorithms proposed in this study might be simpler and more straightforward, thereby enabling wide utilization across various types of micromotion sensors.

We analyzed heartbeat signal as well as respiration and evaluated the association between Fcv and AHI_TRT_, but the correlation was modest. The disparity between ECG R-R intervals and ballistocardiogram heartbeat intervals may contribute the lower correlation of Fcv. In fact, the ECG-Fcv demonstrated a closer correlation with AHI_TRT_ than Fcv. Another potential factor for this difference might be the greater influence of PLM on ballistocardiogram CVHR than on ECG CVHR. PLM is known to be associated with CVHR, and differentiating CVHR caused by PLM from CVHR due to sleep apnea/hypopnea is generally challenging [7]. After excluding subjects with a PLM index of 15 or higher, the correlation between Fcv and AHI_TRT_ improved, but was still lower compared to that between REI and AHI_TRT_. REI was not affected by PLM, and when combined with REI, Fcv had no significant predictive value for AHI_TRT_. In terms of sleep apnea detection using the micromotion signal, the analysis of the Fcv may not be critical. However, the analysis of heartbeat and body movement would be valuable in evaluating sleep quality, as supported by earlier studies [13, 23, 24].

Finally, regarding HSAT devices, piezoelectric rubber sheet sensors have excellent characteristics: small size, light weight, and thin profile. These features would improve not only the ease of incorporating the sensor into bedding but also portability and practicality, reduce the burden of transfers between beds and patient rooms, and facilitate lending from the clinic to patients. Given the significant variability in sleep apnea severity from night to night [18], prolonged monitoring over multiple nights in a home setting is desirable to accurately assess the disease's characteristics in real-world conditions. In such scenarios, the small and lightweight design of the device becomes crucial for convenient handling and ensuring effective maintenance of cleanliness and hygiene.

This study has several limitations. First, the participants were patients who required polysomnography, and the pretest probability of moderate-to-severe sleep apnea was 41% in the test group. When applying to a population with a lower probability, it is expected that the positive predictive accuracy will decrease accordingly. Second, while the sheet sensor was uniformly positioned from the subject's axilla to the lower end of the sternum, there is a possibility for further optimization of its placement for more effective detection of sleep apnea–hypopnea. Third, due to the limited number of cases, the analysis of the effects of comorbidities and of the type of sleep apnea (obstructive, central, or mixed) on the classification performance were not conducted. Fourth, differences in sleep apnea–hypopnea detection effectiveness by sleep stage could not be evaluated because they are disturbed by large changes in respiratory amplitude due to body posture/position. Fifth, the combined predictive value of Fcv and REI may be further improved by machine learning approaches, but they also require a larger sample size. Lastly, the study was conducted in a sleep laboratory, but the product is intended for home use. Future researches are needed to address these limitations and investigate the applicability of the findings in a home setting.

## Conclusions

This study demonstrates that the analysis of the respiratory component, detected using piezoelectric rubber sheet sensors to measure REI, provides a valuable method for HSAT. The optimized REI closely correlated with the AHI_TRT_, displaying strong sensitivity and specificity in identifying subjects with varying severity of sleep apnea–hypopnea. Additionally, while the Fcv showed some correlation with AHI_TRT_, its incorporation did not enhance the predictive capability of the regression model when combined with REI. These findings suggest that the analysis of the respiratory component using piezoelectric rubber sheet sensors presents a promising approach for HSAT, offering a practical and effective means of estimating sleep apnea severity.

## Figures and Tables

**Fig. 5:**
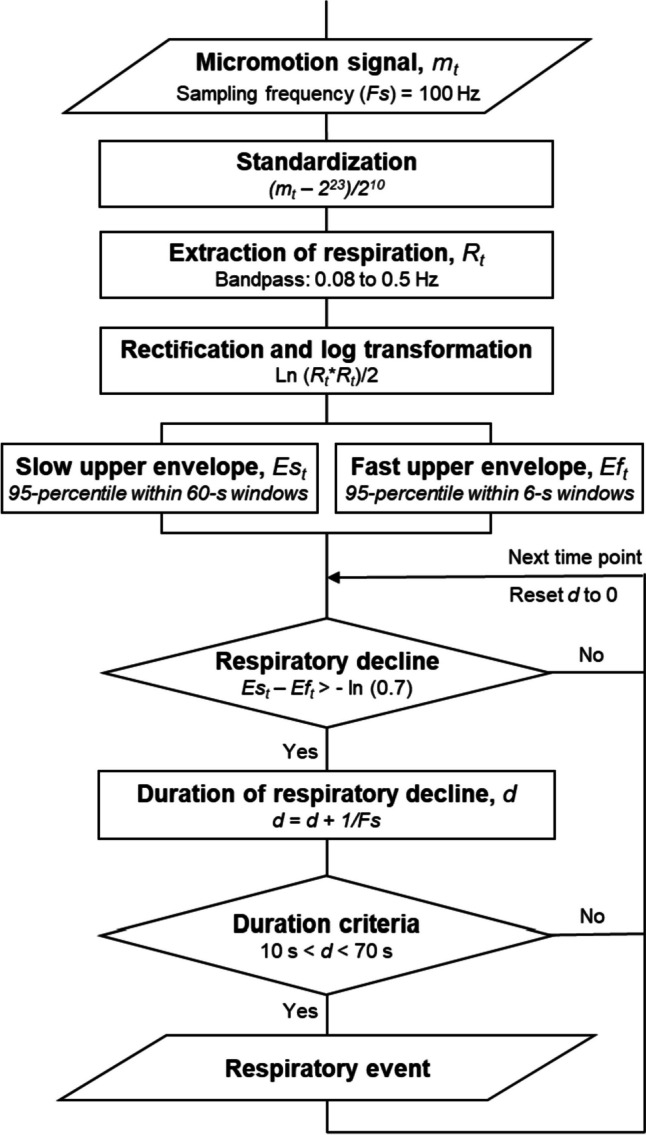
Algorithm developed for detecting respiratory events from micromotion signal of the piezoelectric rubber sheet sensor. ln = natural logarithm

**Fig. 6:**
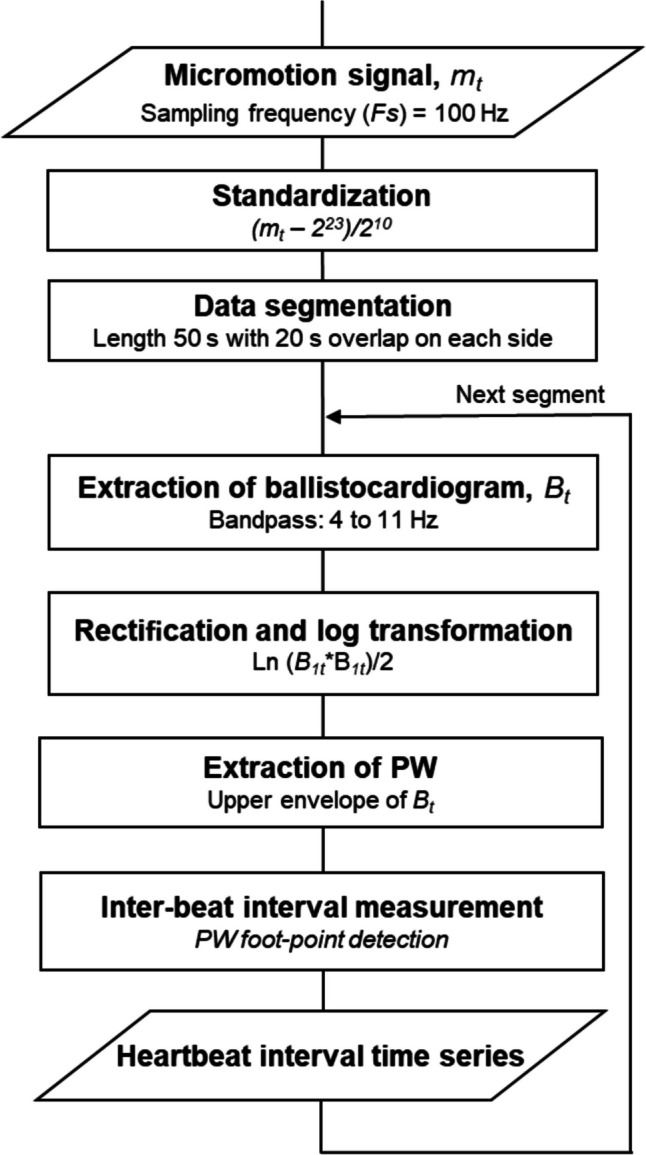
Algorithm developed to obtain heartbeat interval time series from the micromotion signal. Ln = natural logarithm; PW = pulse wave

## Data Availability

The datasets generated during and/or analyzed during the current study are available from the corresponding author on reasonable request and with permission of Gifu Mates Sleep Clinic.

## Appendix

### Detection of respiratory events from respiratory component of micromotion

Output from the piezoelectric rubber sheet sensor included respiratory, body movement, and ballistocardiogram components, which could be separated from each other by the typical frequency bands in which they reside. We developed algorithms to estimate respiratory event index (REI) from the respiratory signal.

The algorithm for detection of respiratory events and calculation of REI is shown in Fig. 5. First, the micromotion signal was standardized to have a dynamic range of ± 2^23^ and the respiration signal was extracted with a 0.08 Ha to 0.5 Hz bandpass filter. Second, the respiratory signal was rectified and natural-logarithmic transformed to reflect the magnitude of respiratory movement while minimizing the impacts of large changes in respiratory amplitude and of large noises. Third, the slow and fast upper envelopes of the respiratory signal were calculated as the moving 95th percentile with window widths of 60 and 6 s, respectively. The slow envelope reflects the average submaximal magnitude of respiratory motion smoothed out by the effects of sleep apnea/hypopnea, while the fast envelope faithfully reflects the changes in the magnitude of respiratory movements including those with sleep apnea/hypopnea. Fourth, a respiratory event was considered to occur when the dip of the fast envelope, compared to the slow envelope, surpassed a threshold value (ln (1.43)), indicating a 30% decline in magnitude, continuously for a duration ranging from 10 to 70 s. Finally, the hourly frequency of respiratory events during total recording time (TRT) of the polysomnogram was calculated as the REI.

The threshold values for the dips of fast envelope and the duration of dips were determined in the training group using a grid search method. This involved repeated analyses with small parameter adjustments until the closest correlation between REI and AHI was achieved (see main text).

### Measurement of heartbeat interval from ballistocardiogram component of micromotion

The algorithm for measuring heartbeat intervals from the micromotion signal is shown in Fig. 6. First, since the micromotion signal was digitalized by a 24-bit converter, the offset was removed by subtracting 2^23^ and dividing by 2^10^ to standardize the dynamic range to ± 2^13^ (± 8192). Second, the data were divided into segments of 50-s duration with 20-s overlap at both ends. Second, ballistocardiogram was extracted with bandpass filters of 4–11 Hz. The signal was rectified and natural-logarithmic transformed, and pulse wave signals were extracted as the upper envelope of each signal. Finally, the heartbeat intervals were measured as the intervals between successive foot points of the pulse wave signal.

## Abbreviations

*AASM*: American Association of Sleep Medicine
*AHI*: Apnea–hypopnea index
*AI*: Apnea index
*AUC*: Area under the curve
*BMI*: Body mass index
*CAI*: Central apnea index
*CI*: Confidence interval
*CPAP*: Continuous positive airway pressure
*ECG*: Electrocardiography
*Fcv*: Frequency of cyclic variation of heart rate
*HI*: Hypopnea index
*HSAT*: Home sleep apnea test
*IQR*: Interquartile range
*MAI*: Mixed apnea index
*NPA*: Negative predictive accuracy
*OHI*: Obstructive apnea index
*PC*: Personal computer
*PLM*: Periodic leg movement
*PPV*: Positive predictive accuracy
*REI*: Respiratory event index
*ROC*: Receiver operating characteristic
*TRT*: Total recording time
*TST*: Total sleep time
